# Bile acids inhibit equilibrative adenosine transport to alter adenosine receptor signaling in cholestasis

**DOI:** 10.1016/j.jbc.2025.108563

**Published:** 2025-04-30

**Authors:** Arnav Joshi, Sijie Chen, Fazlur Md Rahman, Sreenath Nair, Xiaolin Cheng, Rajgopal Govindarajan

**Affiliations:** 1 Division of Pharmaceutics & Pharmacology, College of Pharmacy, The Ohio State University, Columbus, Ohio, USA; 2 Division of Medicinal Chemistry & Pharmacognosy, College of Pharmacy, The Ohio State University, Columbus, Ohio, USA; 3 Translational Therapeutics, Ohio State University Comprehensive Cancer Center, Ohio State University, Columbus, Ohio, USA

**Keywords:** transporter, adenosine, cholic acid, bile acid, nucleoside, cholestasis, equilibrative, modeling, adenosine receptor, signaling

## Abstract

High plasma bile acid (BA) levels in individuals with cholestasis affect adenosine (Ado) receptor (AdoR) signaling, but the underlying mechanisms are unclear. Here, we investigated BA interference with cellular Ado transport as a putative mechanism for altering extracellular Ado availability for AdoR signaling. Computational modeling and experimental studies revealed that equilibrative nucleoside transporter 2 (ENT2), but not ENT1, is capable of translocating BAs across the mammalian plasma membrane. ENT2-mediated BA transport has low affinity, is pH independent, and is partially sensitive to inhibition by nitrobenzylthioinosine (NBMPR). At cholestatic plasma concentrations of BAs, however, BAs interfere with Na^+^-independent, NBMPR-sensitive, ENTs without affecting Na^+^-driven, NBMPR-insensitive, concentrative nucleoside transporters. Interestingly, this BA interference with ENT transport was largely selective for Ado, with minimal to no impact on the transport of other purine or pyrimidine nucleosides. *Xenopus* oocyte-based studies demonstrated that BA inhibition of Ado transport is in the order ENT3≥ENT2>ENT1, which also corresponds to the intrinsic ability of individual ENTs to transport BAs. *In silico* analysis revealed that Ado and BA tend to occupy similar spaces within the ENT translocation pores and that the polar and hydrophilic pore-lining residues determine the interaction of ENTs with BAs. Furthermore, *in vivo* studies indicated that the accumulation of extraneously administered Ado decreases in the livers of cholestatic mice and that interference with Ado transport alters AdoR signaling. Together, these findings reveal novel ENT-dependent BA‒Ado interactions that may have implications for BA dysregulation of AdoR signaling in cholestatic liver diseases.

Bile acids (BAs), the terminal products of cholesterol metabolism, are known to play essential roles in physiological processes pertaining to lipid digestion, nutrient absorption, and energy metabolism (1, 2, 3). In addition, BAs are known to affect cellular signaling processes. For example, BAs activate nuclear receptor signaling, which involves a family of transcription factors primarily involved in nutrient and xenobiotic metabolism (4). Similarly, BAs impact physiological G-protein-coupled receptor (GPCR) signaling, which regulates carbohydrate and lipid metabolism to maintain metabolic homeostasis (5). Increasing evidence indicates that pathological levels of BAs (≥20–100 μM), such as those observed in the plasma of cholestatic patients, can impair purinergic signaling (6, 7, 8), which is an adenosine-directed GPCR signaling pathway involved in a wide range of physiological functions, such as glandular secretion, the immune response, inflammation, vasodilation, cell proliferation, and wound healing. However, the mechanisms by which high concentrations of BAs impair purinergic signaling are not well understood.

Adenosine receptor (AdoR) signaling is triggered by the interaction of extracellular adenosine (Ado) with one or more of the four P1 purinoreceptors—A1, A2A, A2B, and A3 (9) Depending on the tissue involved and the type of purinoreceptors expressed, Ado could have distinct cellular effects in a concentration-dependent manner (9, 10, 11, 12, 13). For example, the AdoR A1 subtype was shown to participate in the pathogenesis of hepatic fibrosis induced by experimental extrahepatic cholestasis (14), whereas the A2A subtype reportedly elicits anti-inflammatory responses in hepatic pathophysiology (15). These studies also indicate that BA-mediated upregulation of purinergic signaling can be detrimental to tissue repair under cholestatic conditions. Interestingly, A1 receptor deficiency in mice attenuated chemically induced cholestatic liver injury (8, 14), suggesting that the regulation of BA-Ado signaling interplay could play crucial roles in both regulating tissue homeostasis and restoring tissue damage.

Ado-dependent purinergic (adenosinergic) signaling is generally activated in two ways: a) increased release of nucleotides such as ATP, ADP, and AMP (16) which are further hydrolyzed by ectonucleotidases to increase extracellular Ado levels for increased AdoR occupancy or b) inhibition of transporter-mediated cellular uptake and clearance of preformed Ado, leading to the accumulation of extracellular Ado (ligand) that triggers the purinoreceptors (17). Experimental evidence shows how BA-mediated stimulation of purinergic signaling through increased ATP release occurs in exocrine pancreatic cells to increase the intracellular Ca^2+^ concentration (6), which is an integral part of insulin secretion (6, 18). However, evidence as to whether BAs can prevent cellular nucleoside uptake to increase the extracellular Ado concentration and, consequently, purinergic signaling alteration is limited. Furthermore, whether BAs interfere with nucleoside uptake to increase the extracellular Ado concentration is ambiguous (19). In this context, the changes in the transport of Ado and the resulting fates due to alterations in BA disposition remain poorly explored.

Recent studies revealed that BAs can interfere with specific transport processes, which may be relevant for increasing ligand-directed receptor signaling. For example, unconjugated BAs and secondary BAs reversibly inhibit the uptake of long-chain fatty acids through the fatty acid transporters FATP2 and FATP5 in mouse livers and cell lines (20). Since fatty acids can act as secondary messengers involved in the transduction of external signals, any interference of BAs with the transport of such fatty acids can alter their extracellular concentrations transiently increasing their binding to plasma membrane receptors, substituting for the classical second messengers of the inositide phospholipid (IP3) and the cAMP signal transduction pathways (21). This phenomenon has been increasingly studied, with evidence indicating the activation of fatty acid GPCRs such free fatty acid receptor (FFAR) 1 and FFAR4 in pancreatic beta cells, adipocytes, myocytes, and several other cell types (22). Analogously, the cellular uptake of Ado is almost exclusively mediated by specialized nucleoside transporters, *viz.* equilibrative nucleoside transporters (ENT/*SLC29*) and concentrative nucleoside transporters (CNT/*SLC28*), but the impact of BAs on these transport systems and therefore purinergic signaling is understudied. While both families are solute carrier (*SLC*) nucleoside transporters, ENTs are low-affinity and high-capacity transporters that shuttle nucleoside substrates bidirectionally through facilitated diffusion, and CNTs are high-affinity and low-capacity transporters that provide unidirectional influx of nucleoside substrates, including Ado, in an Na^+^-dependent manner. The ENT and CNT transporter families each comprise three subfamilies, ENT1 through ENT3 and CNT1 through CNT3, respectively (23).

Here, we describe a functional interaction between BAs and Ado within the ENT family. Our findings revealed that Ado transporter ENT2, but not ENT1, is capable of low-affinity, cell surface BA transport and that cholestatic concentrations of BAs can more selectively inhibit ENTs-mediated Ado cellular uptake to modify adenosinergic signaling.

## Results

### Pore analysis distinguishes ENT transport features

**Figure 1:**
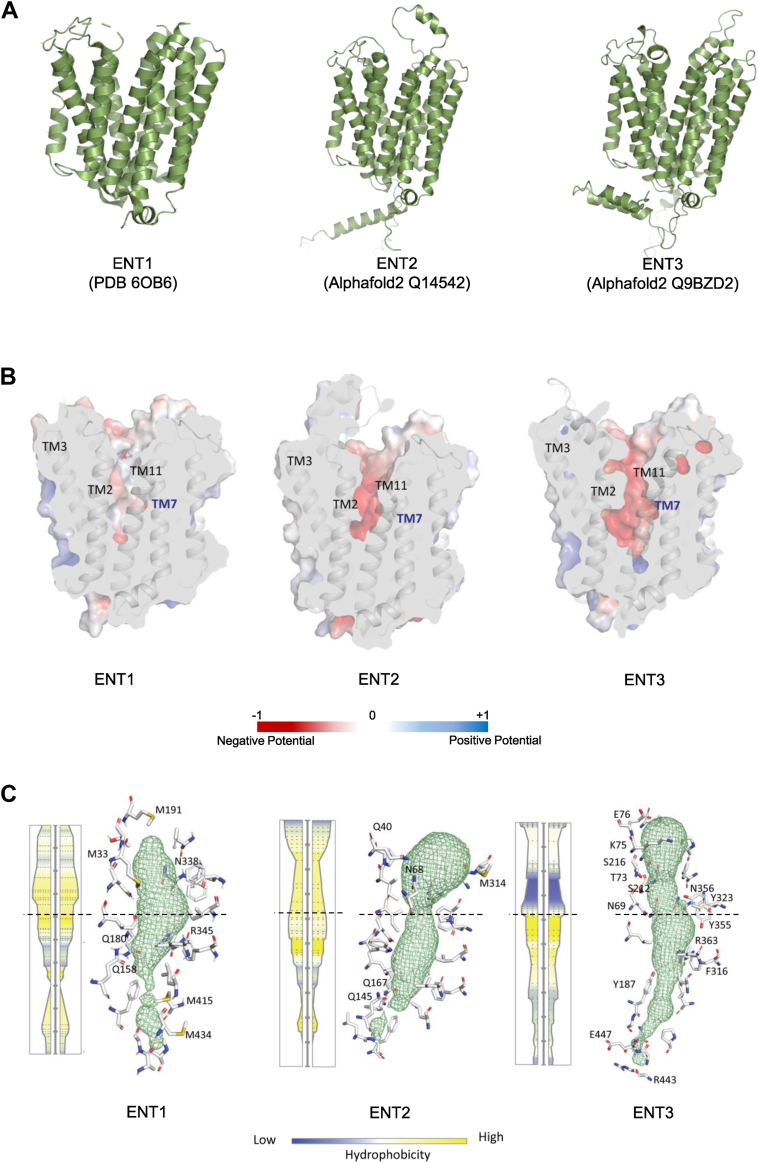
**Structural characterization of human ENTs**. *A*, modeled structures of ENT1 (RCSB PDB 6OB6), ENT2 (Alphafold2 Q14542), and ENT3 (Alphafold2 Q9BZD2). *B*, representation of electrostatic potential mapped on the protein surface for ENT1, ENT2, and ENT3. *C*, pore analysis of ENT1, ENT2, and ENT3 with a schematic diagram representing the hydrophobicity of the pore (on the *left*) followed by 3D visualization of the pore (shown as a *green* mesh) with hydrophilic residues (shown as *ball* and *stick* models) involved; residues from the pore entry (*top*) to the *dashed* line considered for interpretation of hydrophobicity are shown.

We recently reported that lysosome-localized human ENT3 is capable of low-affinity BA uptake (24). Unlike ENT3, whether cell surface–localized human ENT1 or ENT2 can transport BAs is unknown. We modeled the three human ENT transporters by conducting an *in silico* analysis to obtain structural insights into the BA transport characteristics of ENTs. As such, ENT2, but not ENT1, presented greater sequence similarity, identity, and homology with ENT3 (Table S1). We predicted the structures of ENT2 (Alphafold2 Q14542) and ENT3 (Alphafold2 Q9BZD2) (25, 26) *via* Alphafold2 by building a multiple sequence alignment and using the deduced crystal structure of human ENT1 (RCSB PDB 6OB6 https://www.rcsb.org/structure/6ob6) (Fig. 1*A*). Computation of the electrostatic potentials of ENT1, ENT2, and ENT3 using the Adaptive Poisson‒Boltzmann Solver (27) and subsequently mapping them onto the respective ENT protein surface revealed that the central pore of ENTs has a net negative potential, which is the greatest in ENT3, followed by ENT2 and ENT1 (Fig. 1*B*). This result suggested that the pore-lining characteristics of ENT2 are more similar to those of ENT3.

We examined the pore hydrophobicity/hydrophilicity characteristics of each of the ENTs to better understand the detailed pore characteristics (Fig. 1*C*). Computation of the binding pore channel using MOLE (28) revealed that, compared with ENT1 and ENT2, ENT3 has a longer and continuous pore. Like ENT3, where the pore-entry site at the extracellular surface is lined predominantly by the hydrophilic residues E76, K75, S216, T73, S212, N69, Y323, and N356 (six of eight residues are hydrophilic), the pore-entry site of ENT2 is lined with the residues Q40, N68, and M314 (two of three residues are hydrophilic). However, the hydrophilic features of ENT1 are limited, as it is lined by M191, M33, and N338 (only one of three residues is hydrophilic). The overall reduced hydrophobicity of the channel entry site possibly enables interactions with polar substrates and could explain the promiscuous nature of ENT3 in transporting diverse cargos, including BAs (24, 29, 30). Thus, the structural analysis of the ENT transporter proteins along with pore characterization identified similarities and differences among ENTs and revealed that ENT2 is more similar in terms of its pore characteristics to ENT3 than ENT1, likely playing a role in cell surface BA transport.

### ENT2 but not ENT1 transports BAs into oocyte

**Figure 2:**
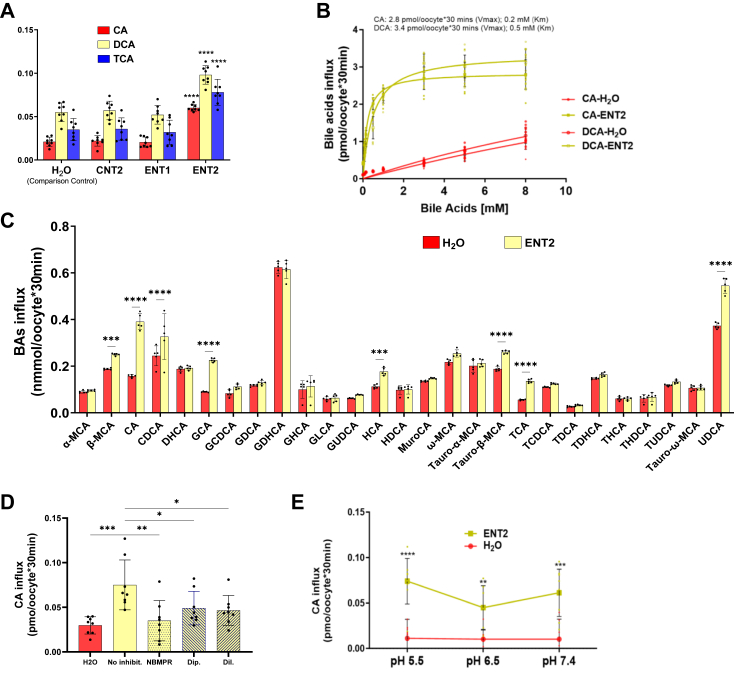
**ENT2, but not ENT1, is capable of transporting BAs.***A*, transport activities of CNT2, ENT1, and ENT2 for different BAs at pH 7.4. *B*, kinetic properties of ENT2 in transporting CA and DCA. *C*, transport activities of various BAs by ENT2 at pH 7.4 measured using LC‒MS-MS. *D*, inhibition of CA influx in the presence of 100 μM NBMPR, dipyridamole, or dilazep in ENT2-injected oocytes (pH 7.4). *E*, pH-independent transport of BA by ENT2. The data in the figures represent the mean ± SD (n = 5–10 oocytes). A representative experiment from 3 to 5 independent experiments is presented. Statistical analysis was performed using two-way ANOVA with Bonferroni’s multiple comparisons; ∗*p* < 0.0332, ∗∗*p* < 0.0021, ∗∗∗*p* < 0.0002, and ∗∗∗∗*p* < 0.0001.

We used a *Xenopus* oocyte-based transport assay that was previously used to examine BA transport by plasma membrane–directed ENT3 with a truncated N-terminus (that harbors lysosomal targeting motif) to experimentally verify whether ENT2 is capable of translocating BAs across the cell membrane (24). We tested both ENT1 and ENT2 along with CNT2, a purine nucleoside-specific concentrative transporter, to investigate their capacities to transport radiolabeled BAs. *In vitro*–transcribed capped mRNAs corresponding to ENT1, ENT2, and CNT2 were injected into processed *Xenopus laevis* oocytes, and the transport of tritiated cholic acid (^3^H-CA), deoxycholic acid (^3^H-DCA), and taurocholic acid (^3^H-TCA) was measured in Na^+^-containing buffer (as described in the Experimental procedures section). Notably, this buffer allows both ENTs (which do not require Na^+^ for transport) and CNTs (which require Na^+^ for transport) to function. The expression levels of each of the ectopically expressed ENTs were similar as determined from quantitative PCR analysis of oocyte extracts using gene-specific primers. Transport was measured at pH 7.4, which is the closest pH to the pH of the extracellular fluid in which cells are exposed to solutes such as BAs to conduct plasma membrane transport (29). Our results revealed that although ENT1 and CNT2 were incapable of transporting CA, DCA, and TCA, ENT2-injected oocytes presented 2.85-, 1.78-, and 2.23-fold increased transport activities for CA, DCA, and TCA, respectively, compared with H_2_O-injected oocytes (Fig. 2*A*). Previous studies demonstrate a low affinity transport of BAs by ENT3 (CA, *V*_max_: 3.07 ± 0.04 pmol/30 min/oocyte, *K*_m_: 167.5 ± 13.3 μM; DCA, *V*_max_: 3.4 ± 0.06 pmol/30 min/oocyte, *K*_m_: 269.6 ± 24.3 μM) (24). The kinetic studies of ENT2 transport of BAs here revealed that the *V*_*max*_ and *K*_*m*_ were 2.84 pmol/oocyte∗30 min and 195 μM, respectively, for CA and 3.359 pmol/oocyte∗30 min and 491 μM, respectively, for DCA (Fig. 2*B*). These experimental findings, along with the structural insights shown in Figure 1, confirmed that ENT2 and ENT3 have similar kinetic characteristics for BA transport, with the former being a cell surface transporter and the latter being a lysosomal transporter.

We next asked whether ENT2 can transport BAs other than the CA, DCA, and TCA. Therefore, we studied several different BAs and used a liquid chromatography–tandem mass spectrometry (LC‒MS/MS) method to quantify the transport activity of H_2_O-injected and ENT2 mRNA-injected oocytes using TCA as a positive control. Our studies indicated significant transport of beta-muricholic acid, CA, chenodeoxycholic acid (CDCA), glycocholic acid (GCA), hyocholic acid, tauro-beta-muricholic acid, TCA and ursodeoxycholic acid (*p* < 0.001 compared with water-injected oocytes) using this assay, confirming the results obtained with the previous radiolabeled assay (Fig. 2*C*). Next, we asked whether the transport patterns of the above BAs mediated by ENT2 are similar to those of nucleoside transport in terms of pharmacological inhibition or pH-dependent properties. Compared with ENT1, ENT2 has less sensitivity to classical nucleoside transporter inhibitors such as nitrobenzylthioinosine (NBMPR), dipyridamole, and dilazep (k_i_: ∼ 10 nM); nevertheless, the transport activity of ENT2 can still be inhibited by higher concentrations of the inhibitors (k_i_: ∼ 10 μM). Hence, we tested whether the transport of BAs by ENT2 can be inhibited by the three inhibitors at 10 μM. Our results revealed that the transport of BAs was partially inhibited by all three nucleoside transporter inhibitors, which reduced ^3^H-CA transport by ENT2 by 53.38%, 34.57%, and 38.02% (values were estimated after NBMPR inhibition) at pH 7.4 compared with the uninhibited control (Fig. 2*D*). We subsequently tested the ability of ENT2 to transport BAs at different physiological pH values ranging from pH 5.5 to pH 7.4 by varying the pH of the transport buffers in which the assay was conducted. The ^3^H-CA transport mediated by ENT2 was not dependent on pH within this pH range (like ENT2-mediated nucleoside transport (29)), as the transport activity was retained under all three conditions (Fig. 2*E*). Thus, the BA transport of ENT2 followed a similar pattern of inhibitor sensitivity and pH independence as that of nucleoside transport.

### BAs inhibit equilibrative Ado transport

**Figure 3:**
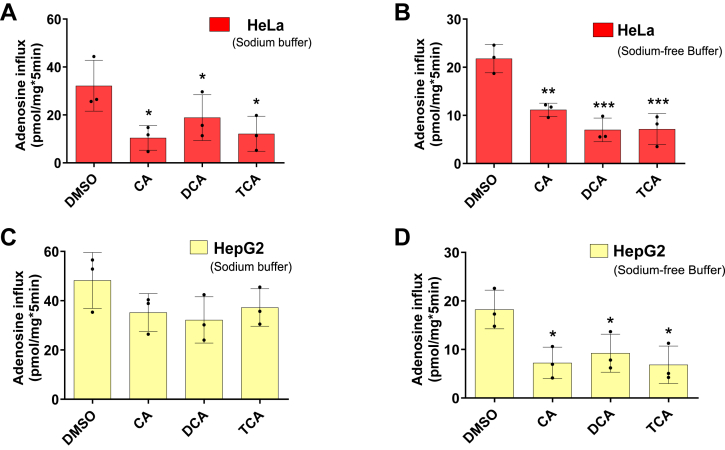
**BAs inhibit ENT-dependent but not CNT-dependent Ado transport in the mammalian cells.***A*–*D*, uptake of 3H-Ado (0.02 μM) in the HeLa and HepG2 cell lines in the presence or absence of different BAs (100 μM) (*A* and *C*) in sodium buffer and (*B* and *D*) in sodium-free buffer. The bars represent the mean ± SD (n = 3). A representative experiment from 3 to 5 independent experiments is presented. Statistical analysis was performed using one-way ANOVA with Dunnett’s multiple comparisons test; ∗*p* < 0.032, ∗∗*p* < 0.0021, ∗∗∗*p* < 0.0002.

In the presence of multiple substrates with similar affinities, the abundant substrate molecules can compete for binding to the transport sites, reducing the transport of the limiting substrate molecules (31). The current findings that cell surface ENT2 transports both nucleosides (K_m_ for Ado transport is ∼100 μM) and BAs (K_m_ for CA transport is ∼195 μM) further prompted us to explore whether relatively high concentrations of BAs (such as that seen in the plasma of cholestatic patients) can inhibit cell surface nucleoside transport. We tested the possibility of BA-mediated nucleoside transport inhibition using a HeLa cell line (expressing ENTs (ENT1, ENT2) (32)) and a HepG2 cell line (expressing both ENTs (ENT1, ENT2) and CNTs (CNT1, CNT2) (33)) to conduct Ado uptake inhibition assays in Na^+^-containing (where both ENTs and CNTs are capable of transport) and Na^+^-free (only ENTs are capable of transport) buffers (Fig. 3 and Table 1). Interestingly, in the HeLa cell line, Ado uptake was inhibited by BAs at a concentration of 100 μM (approximately within the K_m_ range for ENT2 transport of BA) in both Na^+^-containing and Na^+^-free buffers (Fig. 3, *A* and *B*). TCA was most effective at inhibiting Ado uptake in Na^+^-containing and Na^+^-free buffers, with percent uptake inhibition values of 59.20 and 67.21%, respectively (Table 1). On the other hand, in the HepG2 cell line, the inhibition of Ado uptake by BAs was significant only in the Na^+^-free buffer (and not the Na^+^ -containing buffer), in which ENTs alone were functional (Fig. 3, *C* and *D*). In the Na^+^-free buffer, TCA was more effective than CA and DCA, where 62.36% Ado uptake was inhibited (Table 1). Taken together, these data obtained from HeLa and HepG2 cells suggest that the inhibition of Ado uptake by BAs is more likely dependent on the activity of ENTs and not CNTs.

**Table 1:** Inhibition of Ado uptake by BAs in HeLa and HepG2 cells

BAs	% Inhibition of Ado uptake			
	HeLa cell line		HepG2 cell line	
	Sodium buffer	Sodium-free buffer	Sodium buffer	Sodium-free buffer
CA	52.17 ± 0.51	48.78 ± 0.52	n.s.	59.86 ± 0.19
DCA	41.33 ± 0.10	67.86 ± 1.67	n.s.	49.36 ± 0.02
TCA	59.20 ± 0.31	67.21 ± 0.08	n.s.	62.36 ± 0.03

**Figure 4:**
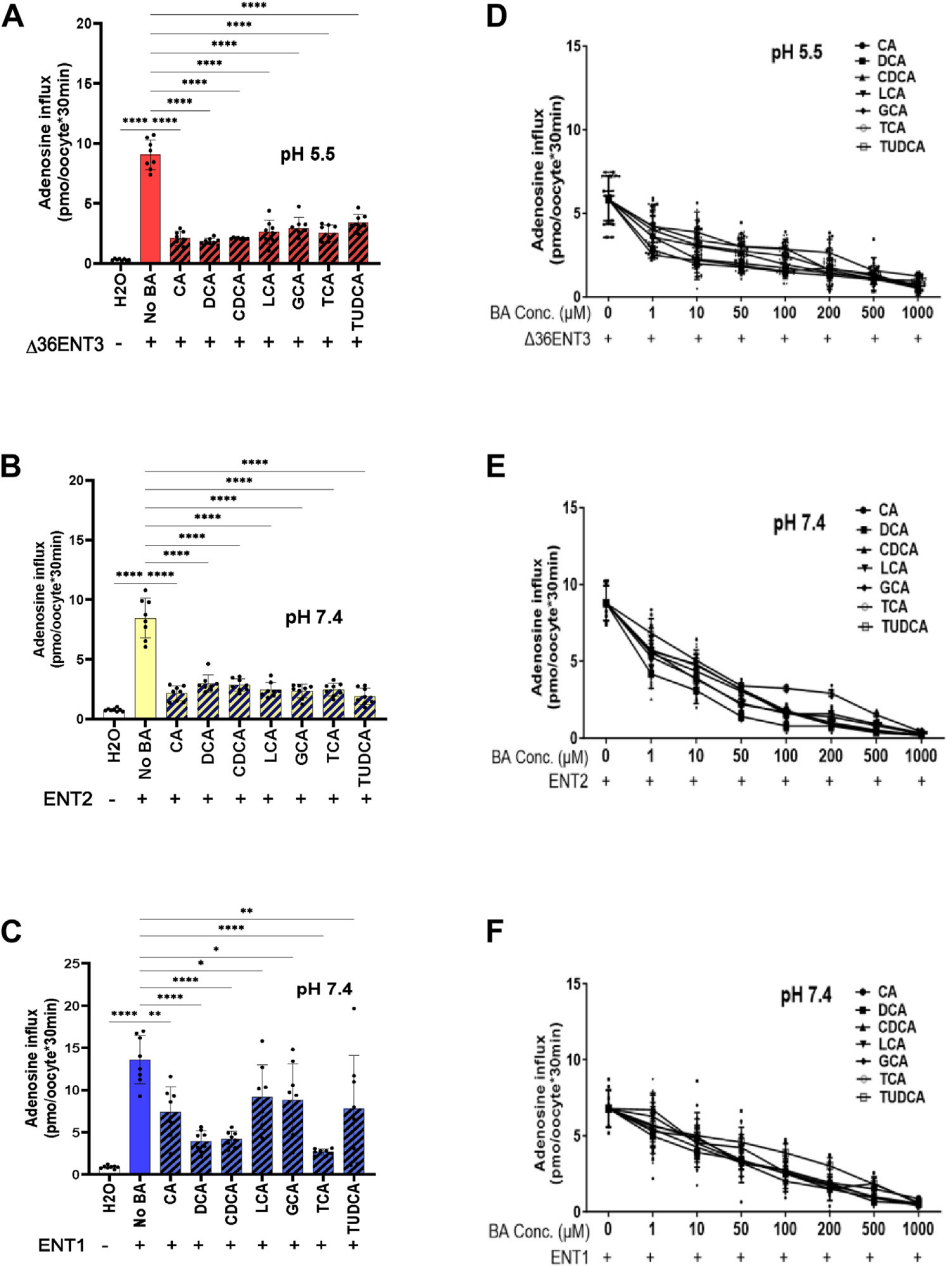
**BAs inhibit Ado transport mediated by various ENTs.***A*–*C*, uptake of ^3^H-Ado (20 μM) into oocytes at 37 °C at 24 h after the injection of ENT transcripts was measured in sodium-free buffer. Inhibition of Ado influx in H_2_O- and ENT-injected oocytes by treatment with different BAs at 100 μM. *D*–*F*, concentration-dependent inhibition of Ado influx in ENT-injected oocytes by different BAs. IC_50_ values of BA inhibition of Ado transport by ENTs indicated in Table 2. The bars represent the mean ± SD (n = 8–12 oocytes). A representative experiment from 3 to 5 independent experiments is presented. Statistical analysis was performed using one-way ANOVA with Dunnett’s multiple comparisons test; ∗*p* < 0.032, ∗∗*p* < 0.0021, ∗∗∗∗*p* < 0.0001.

We expressed individual ENTs (ENT1, ENT2, and ENT3) in *X. laevis* oocytes (as described above) and analyzed the Ado influx from each of the ENTs in the presence of different BAs to better understand whether the inhibition profiles differ across human ENTs. We conducted a radiolabel inhibition assay in which the ^3^H-Ado transport activities of each ENT were separately measured in the presence and absence of different BAs transported by ENT2—CA, DCA, CDCA, LCA, GCA, TCA, and UDCA (Fig. 4, *A*–*C*). We also conducted an analysis of the concentration-dependent inhibition of Ado transport to estimate the IC_50_ values of the BAs (Fig. 4, *D*–*F* and Table 2). These studies demonstrated that among all ENTs, ENT3 was the most sensitive to inhibition by BAs. Among the BAs, CDCA was the most effective at inhibiting ENT3-mediated transport, as it inhibited 77% of adenosine influx, with the lowest IC_50_ of 0.3 ± 0.1 μM, followed by DCA, which inhibited 79% of uptake, with an IC_50_ of 0.6 ± 0.1 μM. ENT2 was most sensitive to DCA, where uptake was inhibited by 65%, with an IC_50_ of 0.9 ± 0.2 μM; tauroursodeoxycholic acid (TUDCA), with an IC_50_ of 4.9 ± 1.1 μM, was able to inhibit 77% of Ado transport. DCA inhibited Ado uptake by 69% in ENT1, with an IC_50_ of 24.3 ± 7.0 μM, followed by GCA (35.05%), with an IC_50_ of 39.5 ± 8.5 μM. Overall, ENT1 appeared to be the least sensitive to BA inhibition among the three ENTs with the highest IC_50_ values (Fig. 4 and Table 2). We expressed CNT2 in *X. laevis* oocytes and assessed Ado uptake after exposure to a fixed concentration of BAs (100 μM) to further confirm that this phenomenon was specific to ENTs. These observations indicated that none of the examined BAs significantly inhibited Ado transport activity mediated by CNT2 (Fig. S1).

**Table 2:** Inhibition of Ado uptake by BAs in *Xenopus* oocytes expressing ENT1, ENT2, or ENT3

Bile Acid	ENT1		ENT2		ENT3	
	% uptake inhibition	IC_50_ (μM)	% uptake inhibition	IC_50_ (μM)	% uptake inhibition	IC_50_ (μM)
CA	45.48	42.1 ± 8.7	74.58	7.1 ± 1.6	77.1	1.2 ± 0.4
DCA	71.01	56.4 ± 14.9	64.77	0.9 ± 0.2	79.9	0.6 ± 0.1
CDCA	69.12	24.3 ± 7.0	66.07	22.9 ± 4.9	77.0	0.3 ± 0.1
LCA	32.59	66.9 ± 15.4	71.04	18.4 ± 4.5	71.2	88.8 ± 27.4
GCA	35.05	39.5 ± 8.5	72.22	16.7 ± 3.9	68.0	10.7 ± 4.6
TCA	80.39	52.9 ± 14.6	70.68	19.5 ± 4.5	72.3	52.6 ± 22.2
TUDCA	42.72	203.5 ± 63.0	77.30	4.9 ± 1.1	62.6	35.3 ± 16.4

### BA inhibits ENTs in an Ado-specific manner

**Figure 5:**
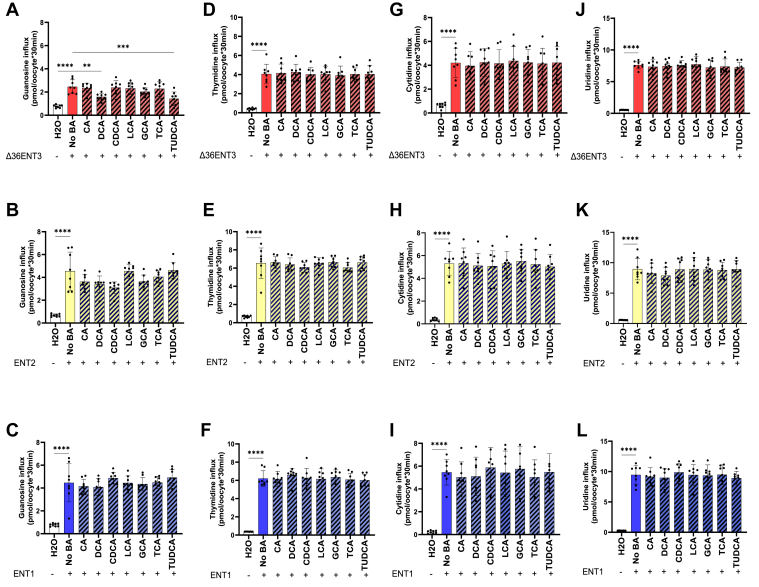
**BA-mediated inhibition of ENT transport activity was largely Ado-specific.***A*–*L*, uptake of ^3^H-guanosine, ^3^H-thymidine, ^3^H-cytidine, and ^3^H-uridine (20 μM) into oocytes at 37 °C after 24 h of injection of ENT transcripts was measured in sodium-free buffers. Inhibition of nucleoside influx in H_2_O- and ENT3-injected oocytes by 100 μM different BAs. The bars represent the mean ± SD (n = 8–12 oocytes). A representative experiment from 3 to 5 independent experiments is presented. Statistical analysis was performed using one-way ANOVA with Dunnett's multiple comparisons test; ∗∗*p* < 0.0021, ∗∗∗*p* < 0.0002, ∗∗∗∗*p* < 0.0001.

Following previous observations of BA-mediated inhibition of Ado uptake, we proceeded to determine whether BAs could inhibit the transport of other endogenous nucleosides. Therefore, we conducted experiments to determine the ability of BAs to inhibit the ENT-mediated uptake of radiolabeled forms of other purine and pyrimidine nucleosides, such as guanosine, cytidine, uridine, and thymidine. Like in previous experiments, we measured the uptake of tritiated nucleosides into *X. laevis* oocytes expressing ENT1, ENT2, or ENT3 (Fig. 5). Surprisingly, our studies on the inhibition of guanosine (another purine nucleoside) uptake indicated that the inhibition of guanosine transport by BAs was lower than that of Ado transport by the BAs transported by ENT3 (25): CA, DCA, CDCA, LCA, GCA, TCA, and TUDCA (Fig. 5*A*). Only DCA and TUDCA inhibited the guanosine transport activity of ENT3 at a concentration of 100 μM BAs (Fig. 5*A*). However, the percent inhibition of guanosine transport mediated by ENT3 (<10%) was much lower than the percent inhibition of Ado transport by ENT3 (>60%) at a concentration of 100 μM BAs (Fig. 5*A*). The analysis of the inhibition of transport activity mediated by ENT2 showed that only DCA and CDCA showed a trend of inhibition of guanosine transport at 100 μM BAs although they were not statistically significant (Fig. 5*B*). No inhibition of guanosine transport mediated by ENT1 was observed with the presence of any BAs at 100 μM (Fig. 5*C*).

Next, we tested whether BAs were capable of inhibiting pyrimidine nucleoside transport mediated by ENT1, ENT2, and ENT3. We tested the abilities of the BAs to inhibit the transport of different tritiated pyrimidine nucleoside (*i.e.*, thymidine, cytidine, and uridine) by ENT3, ENT2, and ENT1 and observed no significant inhibition of pyrimidine nucleoside transport (Fig. 5, *D*–*L*) with DCA inhibition of the ENT2-mediated uridine uptake being the only exception. We also investigated whether BAs inhibited the uptake of therapeutic purine and pyrimidine nucleoside analogs—didanosine (ddI) and zidovudine (AZT), respectively—and observed no inhibition of ddI or AZT transport mediated by ENT1, ENT2, or ENT3 *via* BAs (Fig. S2). Thus, BA inhibitory activity largely appears to be specific to Ado transport. Furthermore, uptake studies in HeLa and HepG2 cell lines revealed that the inhibition of the uptake of guanosine, cytidine, uridine, and thymidine by BAs at a concentration of 100 μM was not significant under Na^+^-free conditions (Fig. S3). The extent of inhibition of guanosine transport (HeLa cells: 16.1% CA, 0% DCA, and 0% TCA; HepG2 cells: 12% CA, 12% DCA, and 17.5% TCA) was much lower than that of Ado transport inhibition (HeLa cells: 48.8% CA, 67.86% DCA, and 67.21% TCA; HepG2 cells: 59.84% CA, 50.6% DCA, and 62.36% TCA) for the same concentration of BAs, confirming that BAs inhibition is specific to Ado transport.

### BAs tend to occupy Ado-binding sites of ENTs

**Figure 6:**
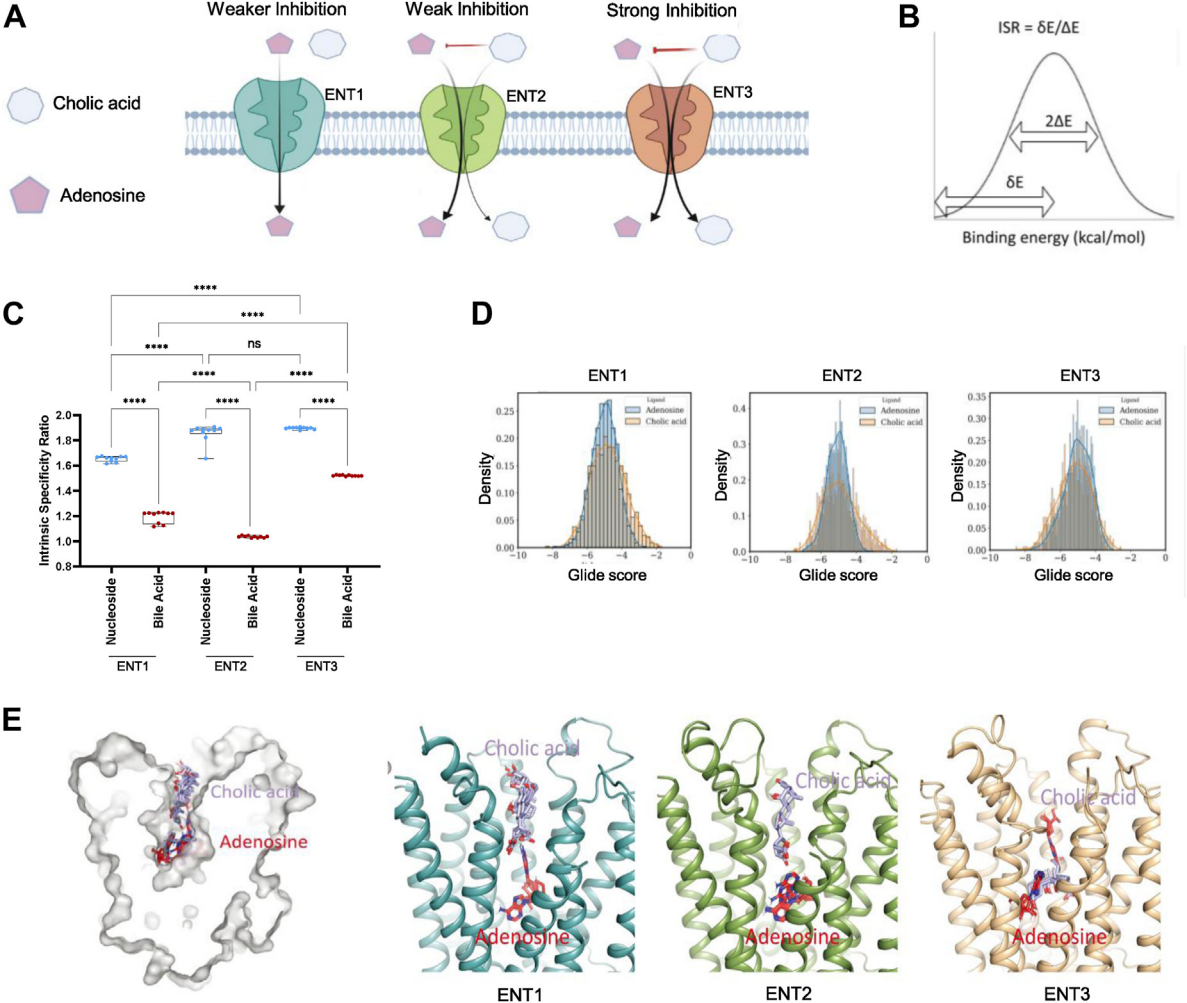
**BAs tend to occupy Ado-binding sites in ENTs.***A*, illustration of the differential ability of CA to inhibit Ado transport across the ENTs. *B*, graph illustrating the calculation used to determine the intrinsic selectivity ratio (ISR) for the ensemble docking analysis. *C*, ISR values for two classes of substrates—nucleosides and BAs—in ENT1 (derived from RCSB PDB), ENT2 (derived from Alphafold2 models), and ENT3 (derived from Alphafold2 models). *D*, docking energy landscape analysis of Ado and CA for three ENTs. *E*, illustration of Ado- and CA-binding positions in the ENT1 structure along with a closer view of the binding sites in ENT1, ENT2, and ENT3. Statistical analysis was performed using one-way ANOVA with Tukey's multiple comparisons test; ∗∗∗∗*p* < 0.0001.

The cargo selectivity for ENTs varies considerably, with ENT1 exhibiting high selectivity for nucleosides and ENT2 exhibiting some additional selectivity for nucleobases and BAs. In addition to nucleosides, nucleobases, and BAs, ENT3 exhibits low-level transport of nucleotides (ATPs), organic cations such as 1-methyl-4-phenylpyridinium (MPP+), and the neurotransmitter serotonin (30). ENT3 also transports many dideoxynucleosides (*e.g.*, AZT, ddI, dideoxycytidine), a class of anti-HIV nucleoside reverse transcriptase inhibitors (30). Thus, ENT1 appears to be the most selective and ENT3 the least selective of all ENTs with respect to cargo selectivity. We next computed the intrinsic specificity ratios (ISRs) of various ENT cargos (as described in Experimental procedures), which included Ado or CA, as substrates to better understand cargo selectivity and gain insights into the molecular mechanisms of differential inhibition of ENT-mediated Ado transport by BAs (Fig. 6*A*). The ISR of a substrate or a class of substrates is calculated by dividing the mean docking energy by the gap docking energy (between the native binding mode and the mean binding mode) of the substrate(s) (Fig. 6*B*). For robust data analysis, we randomly selected 70% of the data points generated from the docking study and repeated the selection process 10 times to construct a final dataset. The results showed that the nucleosides had the highest ISR values, with only smaller variances among the three ENT isoforms (Fig. 6*C*). Interestingly, the BAs had the highest ISR value for ENT3 and had lower values for both ENT1 and ENT2, suggesting the higher binding specificity of the BAs only for ENT3 (Fig. 6*C*).

Furthermore, we also performed multiple molecular dynamics (MD) simulations starting from different conformations or states and collected representative conformations from the resulting MD trajectories to conduct ensemble docking and to account for protein dynamics in docking energy analysis (see Experimental procedures). Two representative substrates, Ado and CA, for nucleosides and BAs, respectively, were docked to the structural ensembles of the three ENTs. We plotted their docking energy distributions, and the results are shown in Figure 6*D*. ENT1 shows a narrower docking energy distribution for Ado than CA. Similarly, in the case of ENT2, Ado shows a narrower docking energy distribution than CA does. This result suggests that Ado binds to ENT1 and ENT2 much more specifically than CA does. However, for ENT3, the docking energy distribution of CA resembles that of Ado, implying that CA may adopt a similar binding mode and thus may more favorably compete with Ado for binding to ENT3 (Fig. 6*D*). We further visualized the most stable states generated by Glide XP docking and observed that CA binds closer to the vestibule near the extramembrane region, whereas Ado binds deeper in the pore (Fig. 6*E*). However, the binding poses of CA and Ado, although not overlapping, tend to cluster together. The probability of CA occupying the Ado-binding site decreases in the order ENT3 > ENT2 > ENT1 (Fig. 6*E*). The different binding poses of ENTs within the Ado translocation pores of ENTs explain the variations observed in the ability of BAs to inhibit Ado transport by the three ENTs. In addition, these analyses support that BAs tend to bind to the Ado-binding sites of ENTs, thereby competitively inhibiting the binding of Ado to the inner pore and leading to reduced transport efficiency.

### Polar/charged residues in the ENT translocation pore anchor BAs

**Figure 7:**
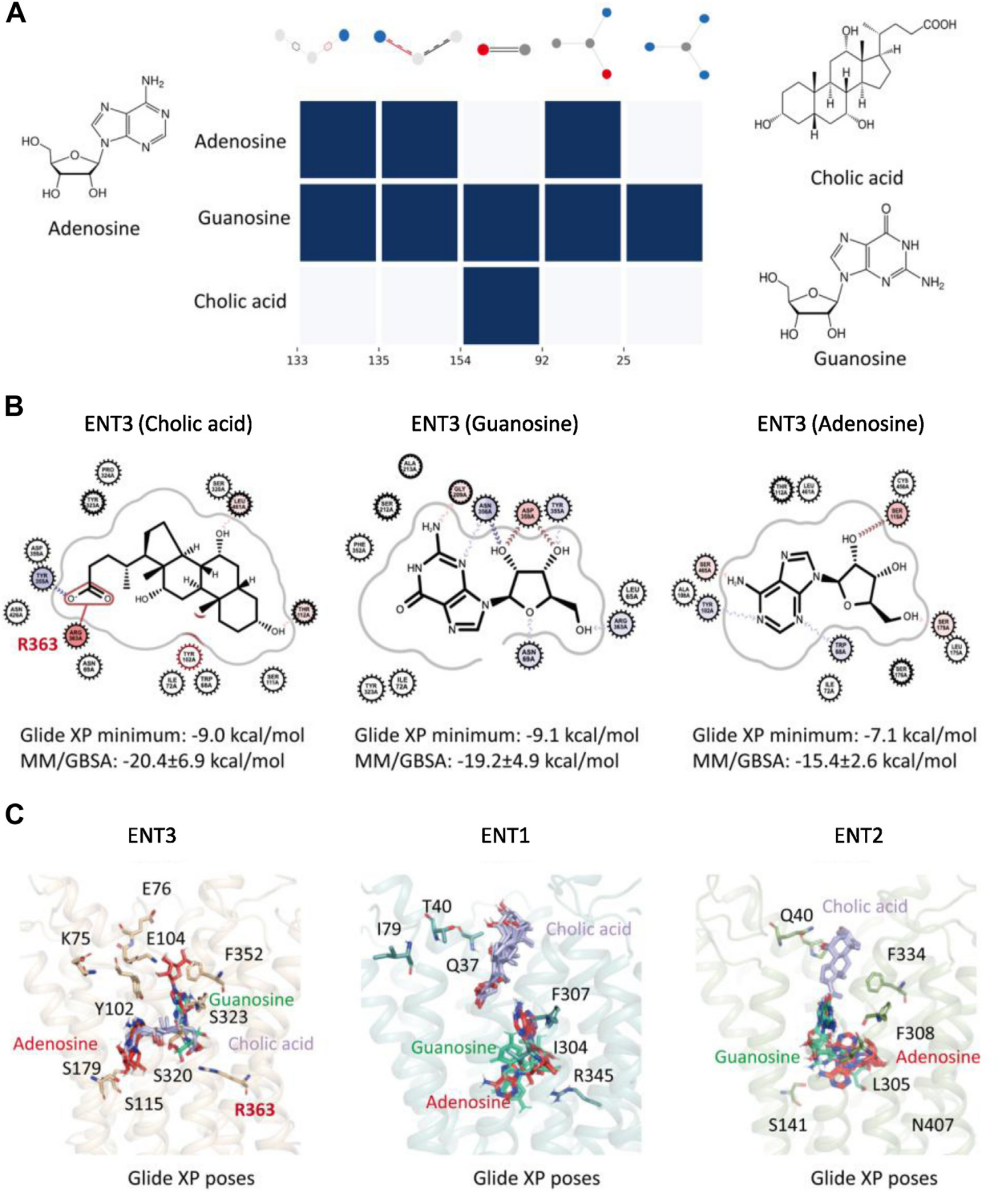
**Molecular mechanisms of BA-mediated ENT inhibition.***A*, substrate feature analysis for Ado, guanosine, and CA, where the *blue*, *red*, and *gray circles* indicate nitrogen, oxygen, and carbon atoms, respectively. The lines indicate bond order connectivity. The central graph represents the MACCS fingerprints characterized from the ML-QSAR model. *B*, 2D plots of Glide XP docking poses of Ado and guanosine in ENT1 (PDB: 6OB7), ENT2 (homology model based on 6OB7), and ENT3 (homology model based on 6OB7). *C*, overlapping binding poses of Ado, guanosine, and CA in all ENTs. Proteins are shown in the cartoon representations for ENT1 (*blue*), ENT2 (*green*), and ENT3 (*wheat*). The interacting residues are in stick representation and labeled. Ado is a *red stick*, and CA is a *purple stick*.

The Ado-specific interaction of BAs, even among purine nucleosides, prompted us to investigate the molecular mechanisms involved in the cargo‒transporter interaction. Since Ado and guanosine are both purine nucleosides that share similar scaffolds, we considered these two molecules as our target substrates of ENT3 for further *in silico* analysis. Moreover, these substrates are sensitive to BA-mediated inhibition to varying extents, with Ado exhibiting greater inhibition than guanosine. We built a machine learning–based quantitative structure–activity relationship (ML-QSAR) model to identify the structural features responsible for the differential inhibitory activities (Fig. 7*A*). We utilized the Molecular ACCess System (MACCS) to establish a correlation between ENT substrate structures and BA substrate-associated activity (34). MACCS encodes the structural features of substrates. Fingerprints determined using this system are able to succinctly represent chemical structures. These fingerprints can then be used to develop computational models that correlate structural features with substrate-associated activities, such as inhibitory activities. For our model, we aimed to correlate the MACCS fingerprints, keys used for measuring molecular similarity, of the 52 known ENT3 ligands, including nucleosides, nucleobases, nucleotides, monoamines, and BAs, with their estimated fold increases in substrate transport. After obtaining a robust model with an accuracy of 0.8, we determined the weights of the individual features contributing to the differences in activity. The ML-QSAR result suggests that the hydroxyl group is a highly important feature among all the MACCS fingerprints that dictates the difference in activity (Fig. 7*A*). This observation is consistent with previous findings that hydrogen bonding involving 3′-OH is essential for CNTs (CNT1 and CNT2) and ENT1 (35).

The above finding highlights the importance of potential hydrogen bonds and polar interactions for solute transport but still does not resolve the question of why guanosine is not inhibited by CA. We identified five features that are not shared among the three ligands to address this question, as shown in Figure 7*A*. The carbonyl group is the only functional group that is common for guanosine and CA but is absent from Ado and could be the key functional group responsible for selective binding to the transporter. We visualized the best docking poses from Glide XP for Ado, guanosine, and CA to understand the transporter‒substrate interactions determined from the ML-QSAR results. The 2D interaction diagram depicts the formation of a salt bridge between CA and R363, the key residue interacting with negatively charged BAs. Docking further suggested that R363 has the potential to interact with the ribose ring of guanosine, which impedes Ado binding (Fig. 7*B*). A lower binding energy implies that the ligand‒protein binding is stronger. The ENT3 docking energies of guanosine and CA are 2 kcal/mol lower than that of Ado, suggesting that ENT3 binds better to CA and guanosine than Ado. We further recorded 1000 snapshots from each 10 ns MD simulation for the Molecular Mechanics, General Born Surface Area energy calculations. The binding free energies of guanosine and CA are 4 kcal/mol lower than that of Ado, which is consistent with the docking results (Fig. 7*B*). Taken together, these results support that BAs can compete for the better binding of Ado but not that of guanosine to ENT3.

Finally, we examined the extracellular loop regions in more detail. The extracellular loop regions formed by the polar/charged residues E75, K75, E104, and Y102 are exclusively present in ENT3, whereas the corresponding residues are Q40 and P37 in ENT2 and T40, Q37, and I79 in ENT1 (Fig. 7*C*). Hence, compared with ENT1 and ENT2, these polar/charged residues in ENT3 are more likely to anchor and concentrate polar substrates, such as BAs, for transport. Additionally, we identified an interesting serine cluster formed by S179, S320, and S115 at the core of the second transmembrane domain of ENT3. A comparison of the residues located at the same positions in ENT2 and ENT3 suggested that this serine cluster is present only in ENT3 (Fig. 7*C*). S320 in ENT3 is substituted by I304 in ENT1 and by L305 in ENT2 (Fig. 7*C*). We observed that the corresponding residue for Y323 of ENT3 is F307 and F308 in ENT1 and ENT2, respectively (Fig. 7*C*). Tyrosine (Y323), which has an additional hydroxyl group, is more likely to enhance the hydrophilic interactions of the ENT3 pore than the phenylalanine residues (F307 and F308) of ENT1 and ENT2. Overall, our ML-QSAR model suggests the role of polar interactions in dictating the differential inhibitory activities of BAs for the transport of Ado and guanosine. The favorability of guanosine binding is most likely due to the carbonyl group. These results are in further agreement with our findings that BAs are competitive inhibitors of Ado transport by ENTs in the order of ENT3>ENT2>ENT1.

### BAs reduce Ado uptake to alter AdoR signaling *in vivo*

**Figure 8:**
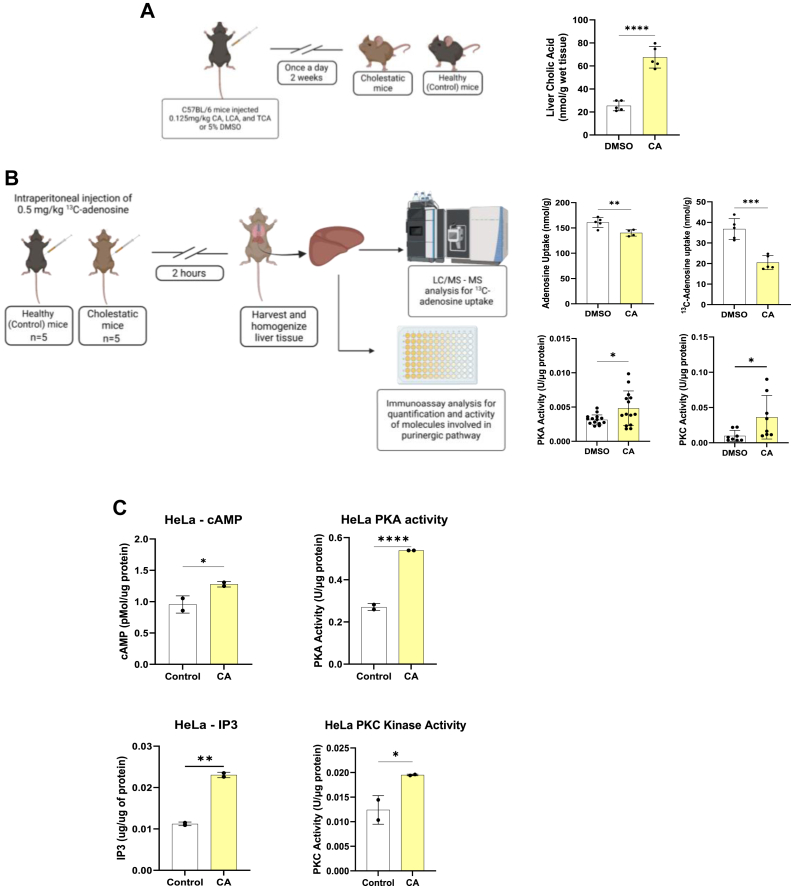
**BA inhibition of Ado transport alters purinergic signaling.***A*, schematic illustrating the generation of a cholestatic mouse model by a once daily i.p. injection of CA, LCA, and TCA (0.125 mg/kg each) over 2 weeks. Graph representing CA levels in the livers of control and generated cholestatic mice. *B*, workflow illustrating the analysis of Ado uptake and signaling in cholestatic mouse livers. Graphs represent endogenous Ado levels in the livers of control and cholestatic mice, uptake of ^13^C-Ado in the mouse liver 2 h after the intraperitoneal injection of 0.5 mg/kg ^13^C-Ado in control and cholestatic mice, and PKA and PKC activities in liver lysates. *C*, BA-mediated Ado transport inhibition increases PKA and PKC signaling activity in HeLa cells. Graphs represent quantification of levels of cAMP and IP3 as well as PKA and PKC activities. The data in (*A* and *B*) are presented as the mean ± SD (n = 5 mice) ∗∗∗∗, *p* = 0.001 (Student's *t* test). *C* and *D*, mean ± SD (n = 7, mice; n = 2 assay duplicates, cells); statistical analysis was performed using two-tailed Student’s *t* test; ∗*p* < 0.0332, ∗∗*p* < 0.0021, ∗∗∗*p* < 0.0002 and ∗∗∗∗*p* < 0.0001.

We directly asked whether high plasma BA levels could inhibit ENT-mediated Ado uptake in the mouse liver (that exhibits high level of expression of ENTs (36)) to investigate whether BA elevation inhibited hepatic Ado uptake, potentially leading to alterations in the Ado disposition *in vivo*. We tested this hypothesis by intraperitoneally injecting CA, LCA, and TCA (0.125 mg/kg each in 5% DMSO) over a period of 2 weeks to establish a cholestatic mouse model. Simultaneously, an equal number of age-matched mice were administered 5% DMSO to develop a control group. Furthermore, we intraperitoneally administered [^13^C]-Ado (adenine riboside-^13^C) to cholestatic and control mice at a concentration of 0.5 mg/kg. The mouse livers were harvested at 2 h postinjection, and the amounts of CA and [^13^C]-Ado were quantified *via* LC/MS-MS. Our study revealed a 2.67-fold increase in the CA level in the livers of mice injected with CA compared with that in the livers of control mice (Fig. 8*A*). Our studies also showed a significant reduction (∼44.39%) in the uptake of [^13^C]-Ado in cholestatic mice compared with control mice (Fig. 8*B*). We also examined the total Ado content (includes endogenous Ado) in the harvested mouse livers using LC‒MS‒MS. Intriguingly, a significant reduction (∼12%) in the total Ado level was observed in CA-injected mice compared with that in control mice (Fig. 8*B*). In combination with our *in vitro* and *in vivo* findings, as well as a recent study showing the inefficiency of BAs in inhibiting thymidine uptake in the mouse liver (37), we conclude that BAs potentially alter Ado metabolism and homeostasis but not that of other nucleosides by controlling their tissue uptake *via* the modulation of ENT function.

High concentrations of BAs that inhibit ENT-mediated Ado transport could lead to an increase in extracellular Ado levels and therefore impact cell surface AdoR signaling. We examined the hepatic lysates of cholestatic mice that exhibited reduced extraneous Ado uptake for changes in AdoR signaling to test our hypothesis that BA-mediated inhibition of cellular Ado transport can modify adenosinergic signaling. Notably, the total hepatic lysates generated from the livers excised from these mice at the end of the study period showed increased activity of PKA, as examined by the phosphorylation of an immobilized PKA substrate in the presence of ATP, using a colorimetric assay (Fig. 8*B*). Along with increased PKA activity, an increased PKC activity was observed when examined with a similar colorimetric assay but using an immobilized PKC substrate (Fig. 8*B*). To further corroborate these results, we treated cultured HeLa cells that predominantly expressed ENTs with 100 μM CA to inhibit equilibrative Ado transport and examine the effects of BAs in AdoR signaling. Compared with the untreated control, ENT inhibition by CA significantly increased the PKA activity in HeLa cells: control 0.27 ± 0.016 U/μg protein; CA 0.54 ± 0 U/μg protein (Fig. 8*C*). When further quantified for the cAMP levels in the total HeLa cell lysate, we observed a significant increase after BA treatment: control 0.96 ± 0.14 pMol/μg protein; CA 1.28 ± 0.04 pMol/μg protein (Fig. 8*C*). We also observed an increase in the PKC activity in CA-treated HeLa cells: control 0.01 ± 0.002 U/μg protein; CA 0.02 ± 0.0001 U/μg protein along with an increase in IP3 levels: control 0.011 ± 0.0004 μg/μg protein; CA 0.023 ± 0.0006 μg/μg protein (Fig. 8*C*). Together, these data suggest that BA-mediated ENT inhibition of Ado increases extracellular levels of Ado, which can alter purinergic responses through PKA-PKC signaling.

## Discussion

ENT proteins play a major role in regulating nucleoside homeostasis. Their function is believed to depend on structural conformational rearrangements to facilitate substrate translocation across the membrane, and recent crystal structure studies have elucidated two distinct inhibitory mechanisms exhibited by ENT1 (38). In this study, we attempt to decipher the interactions between BAs and ENT subfamily members through experimental and *in silico* approaches. Using *Xenopus* oocyte transport assays, mammalian cell-based uptake assays, and mouse-based substrate interaction studies, we report that the potential of high concentrations of BAs to inhibit nucleoside transporters particularly the ENTs. ENTs are sensitive to BA-mediated nucleoside transport inhibition in the order ENT3 > ENT2 > ENT1. However, the transport profiles of CNTs, which do not exhibit BA transport, remain unaffected at these concentrations of BAs. The lack of structural information has hampered the understanding of why BAs have different inhibitory effects on nucleoside transport. We used state-of-the-art protein structural modeling to characterize the various properties of the three human ENT proteins. Our combined homology modeling, docking, and MD simulation results revealed that CA can specifically inhibit Ado transport by occupying the substrate-binding site. Furthermore, our ensemble docking results demonstrate that BAs bind ENT3 more strongly than ENT1 and ENT2.

Ado is a ubiquitous solute that plays an indispensable role in pathophysiological processes. In addition to providing a building block (dATP) for nucleic acid synthesis *via* the salvage pathway and serving as the core of essential cellular metabolites, *for example*, nicotinamide adenine dinucleotide, flavin adenine dinucleotide, and Coenzyme A, Ado plays a crucial role in regulating immune processes and tissue protection through purinergic signaling. The purinergic signaling pathway is nonadrenergic and noncholinergic, driven primarily by the stimulation of the cell surface adenosine GPCRs A1, A2A, A2B, and A3, which regulate several basic cell processes pertaining to proliferation, differentiation, and apoptosis (39), making the Ado receptors and transporters dynamic components of the purinome (23). The high expression of AdoRs enables Ado to modulate physiological functions across different organ systems in an orchestrated manner. Ado is also known to regulate different vascular functions, such as inflammation, neurotransmission, vasodilation, and coronary blood supply, by modulating AdoRs (40, 41). Controlled regulation of Ado metabolism is necessary to maintain an optimal level of Ado to meet the physiological demands of vital organs. However, the regulatory mechanisms of Ado, particularly those governing Ado disposition and their consequences for altered AdoR signaling, are poorly understood. The results of the present study indicated an interaction between cholestatic levels of BAs and Ado uptake mediated by ENTs in cultured cells and mouse tissue, which dysregulates adenosinergic signaling and homeostasis. Further evidence from this study revealed that the inhibition of cell surface ENTs results in Ado accumulation in the extracellular milieu, which leads to altered PKA and PKC signaling (39, 42, 43, 44). It also presents key evidence for how apparently unrelated solutes can interfere with transport activity to relay information to receptors in the cell membrane and impact cell signaling in pathophysiological states.

Our studies support a competitive mechanism of BA inhibition of equilibrative Ado transport. The most abundant BA in the human plasma CA is transported by ENT3 and ENT2 at *k*_*m*_ of 167.5 μM and 195 μM, respectively, which is closer to the estimated *k*_*m*_ of ENT2 for Ado transport (∼100 μM (45)) but much lower than the *k*_*m*_ of ENT3 for Ado transport (2 mM (45)). This suggests that BAs can more readily displace Ado from ENT3-binding pocket than that of in ENT2 as demonstrated here by our computational docking interaction of these solutes. ENT1 has a higher affinity for Ado than CA with independent studies reporting values ranging from 40 to 215 μM, that together with the Ado-CA docking findings here, demonstrated the limited ability of BAs to displace Ado from ENT1-binding sites when compared to those in ENT3 or even ENT2. The estimated k_m_ value for guanosine (2.7 mM) in cloned ENT2-expressing ENT-deficient PK15 cells (46) is much higher than that of CA (∼200 μM) but still fails to explain why the relatively higher affinity BAs cannot displace guanosine from the ENT1-binding pocket like that of Ado. The ENT3’s K_m_ for guanosine is unavailable, but the ENT3 docking and binding free energies of guanosine and CA are found to be lower than that of Ado. This suggests that ENT3 may bind better to guanosine and CA over Ado, partially addressing the strong impact BAs have on Ado transport than guanosine transport. A distinct extracellular loop region, a serine cluster in the core of ENT3 TM2, and the Y323 residue that enhance the hydrophilic interactions of the ENT3 translocation pore further explains the relatively higher specificity of BA anchoring to ENT3 (and not ENT1) necessary for BA transport activity and specific interaction with Ado, providing additional insights into substrate-binding mechanisms of ENTs.

Several reports have described the clinical manifestations arising from irregular purinergic agonism. For example, chronic exposure to Ado leads to fibrosis of the liver, lung, and kidney primarily through the activation of A2A and A2B, triggering cytokine release (47, 48, 49). Similarly, elevated Ado levels can have deleterious effects on the pathophysiology of CNS disorders, autoimmune conditions, diabetes, and cancer (50). These purinergic response–related events during cholestasis can exacerbate the underlying disease and complicate patient care. For example, we previously discovered that the lysosomal membrane-localized nucleoside transporter (*i.e.*, ENT3) functions as a low-affinity BA transporter and that the deletion of this transporter in mice leads to an ∼20-fold increase in BA plasma concentrations resembling cholestatic conditions. In addition to the loss of lysosomal functions (size, pH, protease activity, *etc.*) coupled with aberrant AMPK/mTOR signaling, the high plasma levels of BAs in ENT3 KO mice may further exacerbate purinergic dysregulation. Consistently, ENT3 KO mice exhibit short survival times, severe hematopoietic dysfunction, and breaches of mesodermal tissue integrity (24, 51). Interestingly, mutations in human ENT3 also lead to a spectrum of human genetic disorders (*e.g.*, H syndrome (52, 53), PHID syndrome (54), dysosteosclerosis (55, 56), Rosai-Dorfman Disease (57), *etc.*) with a multitude of symptoms (51, 58, 59). While the mechanisms of occurrence of these varied phenotypes are not fully understood, alterations in Ado-directed purinergic signaling are likely to lead to phenotypes such as autoimmune-like presentations and insulin-independent type II diabetes that rely on normal Ado signaling. Similarly, pregnancy-induced cholestasis occurs in the late stage of pregnancy in humans and is characterized by intense itching, resulting in the production of pruritus. Cholestasis can make pregnancy intensely uncomfortable and pose a danger to the developing baby (60). The exact mechanism of the development of pruritus due to pregnancy-induced cholestasis is not well understood. During pregnancy-induced cholestasis, the plasma BA level can increase to ≥20 μM (61). Our studies showed that at a 20 μM concentration, several BAs are capable of inhibiting Ado uptake by 50%. One of the major side effects of the ENT inhibitor dipyridamole is pruritus (https://www.accessdata.fda.gov/drugsatfda_docs/label/2019/012836s061lbl.pdf, (62)). Therefore, the induction of pruritus due to increased BA levels may result from the fact that BAs are capable of inhibiting ENT-mediated Ado transport and subsequently modulating purinergic signaling. Further studies are expected to elucidate the mechanisms of various cholestatic complications related to AdoR signaling aberrations.

Membrane transporters are essential gatekeepers that regulate the trafficking of solutes across the cellular or organelle membranes. A growing number of transporters are now known to transport substrates that are structurally less related or even unrelated to their well-recognized endogenous substrates, thereby allowing cargos to compete for binding occupancy within the translocation pore. This phenomenon affects the transport of individual molecules, which are observed at supraphysiological solute concentrations within certain specific tissue compartments. More frequently, these interactions involve a diverse class of drug molecules that are also excellent substrates for SLC transporters. The perturbation of Ado transport affecting AdoR signaling due to conditions such as cholestasis studied here is such an example and provides several new insights into the Ado transporter–receptor interplay. First, our studies expand on the substrate repertoire of ENTs by revealing the promiscuity of ENT2 and ENT3 in transporting BAs. Although certain SLC BA transporters have been shown to exhibit differential selectivity in terms of transporting unconjugated and conjugated BAs (63), our current findings revealed no such selectivity for BA transport mediated by ENT2. Both ENT2 and ENT3 conduct low-affinity transport of a subset of BAs, although the inhibitory potential of BAs toward ENT-mediated Ado transport seems broadly specific to all three ENTs. This property of BAs was reflected by increased extracellular accumulation of Ado, which significantly alters AdoR signaling. The additive effects of individual BAs on cholestasis could also compound the problem by rapidly accumulating extracellular Ado for rapid and complete receptor occupancy. The activation of A1 and A3 AdoRs inhibits the activity of adenylate cyclase, whereas the activation of A2A and A2B promotes adenylate cyclase activity, thereby increasing the cytosolic levels of cAMP (64). In response to elevated cAMP, PKA is activated, stimulating the nuclear cAMP-responsive–binding protein that drives transcription factors (23, 65). Furthermore, Ado receptor signaling can increase cytosolic IP3 levels activating the PKC signaling pathway, which in turn could regulate many downstream signaling pathways. Overall, the activation of AdoRs by high concentrations of extracellular Ado alters the purinergic signaling pathway that regulates cell physiology through specific processes (39, 66). Our findings reveal increased PKA and PKC activities in both cultured cells and mouse tissue, which correlate with reduced Ado uptake driven by cholestatic concentrations of BAs. The structural analysis and experimental findings further reveal the dynamics of changes in Ado transport and the specificity of the involvement of ENTs and not CNTs in this process. Because ENTs are ubiquitously expressed compared with their CNT counterparts, whose expression is somewhat restricted to specialized tissues (*e.g.*, GIT, kidneys, and brain), not surprisingly, cholestasis has a widespread effect on tissue homeostasis. Thus, the current study reveals the structure‒activity relationships of ENTs while comparing the structural similarity of Ado and BAs with respect to their transport activity and interference.

In summary, our integrated structural, functional, and binding energy analyses provide molecular-level insights into why and how BAs inhibit Ado transport mediated by Ado transporters, thereby impacting the Ado transporter‒receptor interplay in purinergic signaling. Our findings lay a foundation for future work to not only expand our understanding of the pathophysiology of cholestasis-associated adverse effects but also to possibly identify drugs that restore AdoR signaling homeostasis in cholestatic liver diseases.

## Experimental procedures

### Materials and reagents

Unlabeled BAs were purchased from Sigma, and radiolabeled BAs were purchased from American Radiolabeled Chemicals. ^3^H-Radionuclides were purchased from Moravek Radiochemicals. ^13^C-Ado was purchased from Omicron Biochemicals. Collagenase A was purchased from Roche Applied Science. PMSF, 4′,6′-diamidino-2-phenylindole, uridine, adenosine, gentamycin, tricaine methanesulfonate, and other chemicals were purchased from Sigma. Fetal bovine serum and horse serum were purchased from Corning Life Sciences. Penicillin‒streptomycin was obtained from Invitrogen. BCA protein assay reagent and SuperSignal West Pico Chemiluminescent substrate were obtained from Thermo Fisher Scientific. Taq polymerase, dNTPs, nuclease-free water, and restriction enzymes were purchased from Promega.

### Cell culture and transport assay

HeLa (CCL-2) and HepG2 (HB_8065) were procured from ATCC. Both cell lines were cultured in Eagle's minimum essential medium and Dulbecco's modified Eagle's medium, respectively, supplemented with 10% fetal bovine serum and penicillin-streptomycin (1X). After cells were tested free for *mycoplasma* contamination, they were plated in 24-well plates, and transport assays were conducted when the cells were 80% confluent. The cells were briefly rinsed with PBS, and then the uptake of radiolabeled substrates was measured after a 5 min incubation in sodium containing (130 mM NaCl, 20 mM Tris–HCl, 3 mM K_2_HPO_4_, 1 mM MgCl_2_.6H_2_O, 2 mM CaCl_2_, 5 mM glucose, pH 7.4) or sodium-free (130 mM N-methyl-D-glucamin, 20 mM Tris–HCl, 3 mM K_2_HPO_4_, 1 mM MgCl_2_.6H_2_O, 2 mM CaCl_2_, 5 mM glucose, pH 7.4) transport buffers at 37 °C. Uptake was terminated by washing the cells three times with ice-cold arrest buffer containing 20 mM uridine. The cells were shaken for 30 min in 10% SDS for complete dissolution, after which the radioactivity was quantified with a Beckman liquid scintillation counter. The data are presented as the mean ± SD (n = 3).

### Ado signaling experiments

Confluent HeLa cells were treated with 100 μM CA dissolved in a 10% DMSO vehicle. After being treated for 14 h, the cells were harvested, lysed, and diluted with the appropriate buffers provided in individual kits. cAMP levels were quantified using a complete cAMP ELISA kit (ADI-900-136A) purchased from Enzo Biochem. PKA activity was assessed using the PKA Colorimetric Activity Kit (EIAPKA) supplied by Thermo Fisher Scientific. PKC activity was measured using PKC kinase activity kit (ADI-EKS-420A) purchased from Enzo Biochem. IP3 levels were quantified using an IP3 (Inositol Triphosphate) ELISA Kit (EKF58173) supplied by Biomatik Corporation.

### Plasmid constructs and qPCR analysis

The pOX-Δ36hENT3 *Xenopus* expression construct was described previously (30, 67). The complementray DNA clones of human ENT1 and 2 were purchased from Dharmacon. The complete ORF of ENT1 (amplified using forward primer: 5′-GATTA GTCGAC CCACC ATG ACA ACC AGT CAC CAG-3′ and reverse primer: 5′-GTA TCTAGA TCA CAC AAT TGC CCG-3′) and ENT2 (amplified using forward primer: 5′-TTC AAGCTT CCACC ATGGCGCGAGGAGACG-3′ and reverse primer: 5′-GTA TCTAGA TCAGAGCAGCGCCTTGAAGA-3′) were cloned into pOX *Xenopus* expression vector at the 5′SalI-3′XbaI and 5′HindIII-3′XbaI restriction sites, respectively. The plasmid constructs for ENT1 and ENT2 were verified by Sanger sequencing at the Ohio State Genomic Facility and designated as pOX-hENT1 and pOX-hENT2, respectively.

### In vitro transcription and expression in *Xenopus* oocytes

The generated pOX expression constructs were linearized with either the Not1 or Sac1 restriction enzymes. The digested products were purified *via* phenol‒chloroform extraction. The mRNAs were synthesized using mMESSAGE mMACHINE (Ambion) transcription kits according to the manufacturer’s instructions. The mRNAs were purified *via* lithium precipitation. Oocytes were procured from Ecocyte. Prior to experiments, procured oocytes were defolliculated by treating with collagenase A-OR II solution followed by washing with Barth’s solution. Following microscopic verification, selected oocytes were incubated to recover overnight at 25 °C in a shaker incubator. Fifty nanoliters (400–800 ng/μl) of equal amounts of mRNAs were injected into defolliculated oocytes the next day. Expression of plasmids was analyzed after RNA isolation from oocyte extracts and conducting qPCR analysis with gene-specific primers as described earlier (30). The injected oocytes were incubated at 15 °C for 24 h before transport assays were performed. The uptake of radiolabeled substrates was measured after a 30 min incubation in transport buffer (100 mM NaCl, 2 mM KCl, 1 mM CaCl_2_, 1 mM MgCl_2_, and 10 mM Hepes, pH 7.4) at room temperature. Uptake was terminated by washing the oocytes three times with arrest buffer containing 20 mM uridine. Individual oocytes were shaken overnight in 10% SDS for complete dissolution, and then the radioactivity was quantified with a Beckman liquid scintillation counter. The data are presented as the mean ± SD (n = 8–12 oocytes). A representative experiment from 3 to 5 independent experiments is presented.

### Quantification of BAs using LC‒MS/MS

The mass spectrometric validation of the transport of all metabolites was performed with oocytes using a previously reported method (24). Briefly, the oocytes obtained after treatment with wash buffer were homogenized with 200 μl of extraction buffer (50% methanol containing 0.1% acetic acid) and spiked with CA-d4 (1 μg/ml) and glycocholic acid-d4 (1 μg/ml) as internal standards. After an incubation at 4 °C for 24 h, the samples were centrifuged at 20,000*g* for 10 min, and the supernatants were collected. The samples (10 μl) were injected into a Thermo Scientific Vanquish UPLC system interfaced with a Thermo Scientific TSQ Quantiva triple-stage quadrupole mass spectrometer equipped with an H-ESI ion source. MS detection was conducted in negative ionization mode, and the transitions monitored for each metabolite are listed in Sup. Table S2. The operational mass spectrometric parameters included a capillary voltage of 4.5 kV; sheath gas of 35 arbitrary units; auxiliary gas of 10 arbitrary units; sweep gas of two arbitrary units; ion transfer tube temperature of 350 °C; vaporizer temperature of 450 °C; dwell time of 50 ms per transition; collision energy of 5 V; and collision-induced dissociation gas of 1.5 mTorr. Chromatographic separation was performed on a Kinetex instrument (100 × 2.1 mm; 1.7 μm particle size) from Phenomenex. The mobile phase consisted of solvent A: water (0.1% formic acid) and solvent B: acetonitrile (0.1% formic acid). The flow rate was set at 0.3 ml/min, and the autosampler was maintained at 4 °C throughout the analysis.

### Quantification of endogenous Ado and ^13^C-Ado concentrations using LC‒MS/MS

Adenosine and ^13^C-Ado concentrations were determined in liver tissue samples using a targeted LC‒MS/MS method. The liver samples (∼50 mg) were homogenized with 80% methanol and spiked with 1 μg/ml reserpine as an internal standard. The samples were thoroughly vortexed for 30 s and then centrifuged at 20,000*g* for 10 min. The supernatant was collected in labeled Eppendorf tubes, and 15 μl of each sample was injected into the LC‒MS/MS system. MS detection was performed in positive ionization mode, and the transitions monitored were m/z 268.12 to −136.11 for Ado, 269.12 to −136.1 for Ado C^13^, and 609.39–195.05 for reserpine. The operational mass spectrometric parameters included a capillary voltage of 4.5 kV; sheath gas of 35 arbitrary units; auxiliary gas of 10 arbitrary units; sweep gas of two arbitrary units; ion transfer tube temperature of 350 °C; vaporizer temperature of 450 °C; dwell time of 50 ms per transition, and collision-induced dissociation gas of 1.5 mTorr. The collision energy was set at 20 V for both Ado and ^13^C-Ado, whereas it was set to 38 V for reserpine. Chromatographic separation was performed on a Scherzo SMC18 column (150 × 4.6 mm; 3 μm particle size) from Imtakt. The mobile phase consisted of solvent A: water (0.1% formic acid) and solvent B: acetonitrile (0.1% formic acid), and the flow rate was maintained at 0.5 ml/min. The gradient program used was as follows: 0 min, 95% B; 2 min, 95% B; 10 min, 10% B; 11 min, 95% B; and 17 min, 95% B. The autosampler was maintained at 8 °C throughout the analysis.

### Mouse study

Female C57BL/6 mice (age 10–12 weeks) were intraperitoneally injected with CA, LCA, and TCA at a concentration of 0.125 mg/kg once a day for 2 weeks (68, 69, 70, 71). After developing cholestasis, ^13^C-Ado was injected into the mice at a concentration of 0.5 mg/kg. Two hours after the injection, mouse livers were harvested, and the amount of ^13^C-Ado uptake in the liver was quantified *via* LC–MS-MS. The data in the figures represent mean ± SD (n = 5–8 mice). All the experimental mice used were bred in house. Animal procedures were performed according to protocols approved by the Ohio State University (OSU) IACUC. Mice were maintained at an ambient temperature of 20 to 22 °C and humidity 40 to 60% with a 12-h light/dark cycle and were given free access to standard rodent chow and water. Mice were euthanatized by carbon dioxide asphyxiation with confirmation of death through cervical dislocation.

### Protein structural modeling

The structures of human ENT2 and ENT3 were downloaded from the AlphaFold database under the entry names Q14542 and Q98BZD2 (25). The amino acid sequences of human ENT2 and ENT3 were obtained from the UniProt database (72). Sequence alignment was performed, and the sequences were projected onto the protein structure with ChimeraX (73). Protein electrostatic potentials were computed with Adaptive Poisson‒Boltzmann Solver (27), mapped onto the protein surface, and plotted with PyMOL (74). The channel analysis was conducted with the MOLE online server (28). Homology structures of ENT2 and ENT3 were constructed using MODELLER basic modeling (75). The crystal structures for human ENT1 (PDB: 6OB6 (38) and 6OB7 (38)) were used as templates.

### Molecular docking

Molecular docking was performed using the Glide SP and XP modules of the Schrodinger Maestro suite (76). The crystal structure of human ENT1 (6OB7) and the homologous structures of human ENT2 and ENT3 based on 6OB7 were used for docking. Protein preparation and refinement were performed using a protein preparation wizard. Water molecules and solvents in the crystal structures were discarded, and missing hydrogens were added. Energy minimization was performed to eliminate steric clashes using the OPLS 2005 force field (77). The binding site was specified around the co-crystalized ligand, and the grid was created using the Maestro Grid tool. For each substrate, 250 docking poses were obtained, and the docking scores were computed with standard precision and extra precision. Substrate structures were obtained from PubChem. All the ligands were prepared using Maestro LigPrep. The docking poses were visualized with PyMOL (74) and Openeye Toolkits 2023.1 by Cadence Molecular Sciences. The Glide SP docking scores were analyzed by calculating the ISR which determines the capability of discriminating between native and non-native (weak) poses of ligand–protein interactions (78).

### MD simulations

MD simulations were performed on eight ENT structures, including two X-ray structures of human ENT1 (6OB6 and 6OB7), homology model structures of human ENT2 and ENT3 based on 6OB6 and 6OB7, respectively, and Alphafold2-predicted structures of human ENT2 and ENT3. All MD simulations were performed with the CUDA version Amber18 (79) using the Charmm36 all-atom force field (80). Each protein structure was embedded in an 80∗80 Å^2^ lipid bilayer composed of 34% cholesterol, 23% POPC, 17% PSM, 11% POPE, and 8% POPS. The system was solvated in a TIP3P water box with 22.5 Å padding on top and bottom of the protein, which was neutralized by adding 0.15 M potassium and chloride ions. After minimization, each system was heated to 300 K and equilibrated for 1 ns at a constant pressure of 1 atm. Finally, 10 independent production simulations, each for a duration of 500 ns, were performed for each bilayer system in the NPT ensemble using the MC barostat (81) and Langevin thermostat algorithms (82). The particle mesh Ewald method (83) with a cutoff distance of 12 Å was employed to handle the long-range electrostatic interactions, and the same threshold value was also used for the truncation of the Lennard‒Jones potentials. The hydrogen atoms involved in the covalent bonds were constrained by the SHAKE algorithm (84). The binding free energies of ENT- CA and ENT-Ado were computed using the MMGB/SA method supported by AMBER22. Using the 3-trajectory approach, 300 snapshots from the MD trajectories were considered for MMGB/SA calculations.

## Data availability

All the data supporting the findings of this study are available within the paper and the supporting information.

## Supporting information

This article contains supporting information.

## Conflict of interest

The authors declare that they have no conflicts of interest with the contents of this article.
