# Supplementary material for: Bile acids inhibit equilibrative adenosine transport to alter adenosine receptor signaling in cholestasis
Source: J Biol Chem. 2025 Apr 30;301(6):108563. doi: 10.1016/j.jbc.2025.108563 (PMC12152883; doi:10.1016/j.jbc.2025.108563)
Supplement: Supporting Information [file mmc1.docx]

Bile acids inhibit equilibrative adenosine transport to alter adenosine receptor signaling in cholestasis

Arnav Joshi^#1^, Sijie Chen^#2^, Fazlur Md Rahman^#1^, Sreenath Nair^1^, Xiaolin Cheng^2^, and Rajgopal Govindarajan*^1,3^

^1^Division of Pharmaceutics & Pharmacology, College of Pharmacy, The Ohio State University, Columbus, OH, 43210, USA

^2^Division of Medicinal Chemistry & Pharmacognosy, College of Pharmacy, The Ohio State University, Columbus, OH, 43210, USA

^3^Translational Therapeutics, Ohio State University Comprehensive Cancer Center, Ohio State University, Columbus, OH 43210, USA

Supporting Information

| Fig/Table No. | Title | Page |
| --- | --- | --- |
| Table S1 | Structural analysis and comparison of the similarity of ENT1 and ENT2 with ENT3 | S-1 |
| Table S2 | MS detection for BAs and metabolites after oocyte transport assay | S-2 |
| Fig. S1 | BAs are incapable of inhibiting adenosine transport mediated by CNT2 | S-3 |
| Fig. S2 | BAs were incapable of inhibiting nucleoside analogs transport mediated by ∆36ENT3 and ENT2 | S-4 |
| Fig. S3 | BAs do not interfere with transport of nucleosides except adenosine | S-5 |

|  | **Modeled structure** | **Sequence similarity (%)** | **Sequence identity (%)** | **Homology (%)** |
| --- | --- | --- | --- | --- |
| ENT1 | 6OB6 (RCSB PDB) | 33.07 | 18.44 | 26 |
| ENT2 | Q14542 (Alphafold2) | 34.27 | 19.15 | 51 |
| ENT3 | Q9BZD2 (Alphafold2) | 100 | 100 | 100 |

**Sup. Table 1:** Structural analysis and comparison of the similarity of human ENT1 and ENT2 with ENT3

| **Compound** | **Polarity** | **Precursor**  **(m/z)** | **Product**  **(m/z)** | **Collision**  **Energy (V)** | **RF Lens**  **(V)** |
| --- | --- | --- | --- | --- | --- |
| CDCA | Negative | 391.27 | 391.27 | 5 | 109 |
| UDCA | Negative | 391.3 | 391.3 | 5 | 150 |
| HDCA | Negative | 391.35 | 391.35 | 5 | 150 |
| DHCA | Negative | 401.2 | 401.2 | 5 | 150 |
| α-MCA | Negative | 407.16 | 407.16 | 5 | 150 |
| β-MCA | Negative | 407.17 | 407.17 | 5 | 150 |
| ω-MCA | Negative | 407.18 | 407.18 | 5 | 150 |
| HCA | Negative | 407.19 | 407.19 | 5 | 150 |
| CA | Negative | 407.21 | 407.2 | 5 | 141 |
| GLCA | Negative | 432.3 | 432.3 | 5 | 150 |
| GDCA | Negative | 448.3 | 448.3 | 5 | 150 |
| GUDCA | Negative | 448.31 | 448.31 | 5 | 150 |
| GCDCA | Negative | 448.5 | 448.5 | 5 | 150 |
| GDHCA | Negative | 458.3 | 458.3 | 5 | 150 |
| GHCA | Negative | 464.31 | 464.31 | 5 | 150 |
| GCA | Negative | 464.5 | 464.5 | 5 | 150 |
| TUDCA | Negative | 498.29 | 498.3 | 5 | 195 |
| TDCA | Negative | 498.31 | 498.3 | 5 | 152 |
| THDCA | Negative | 498.33 | 498.33 | 5 | 150 |
| TCDCA | Negative | 498.34 | 498.34 | 5 | 150 |
| TDHCA | Negative | 508.2 | 508.2 | 5 | 150 |
| T-β-MCA | Negative | 514.25 | 514.25 | 5 | 150 |
| T-α-MCA | Negative | 514.27 | 514.27 | 5 | 150 |
| THCA | Negative | 514.28 | 514.28 | 5 | 150 |
| T- ω -MCA | Negative | 514.29 | 514.29 | 5 | 150 |
| TCA | Negative | 514.3 | 514.3 | 5 | 248 |
| THCA | Negative | 514.32 | 124 | 55 | 248 |

**Sup. Table 2: MS detection for BAs and metabolites after oocyte transport assay**

**Sup Fig 01**: Bile acids are incapable of inhibiting adenosine transport mediated by CNT2: Figure represents inhibition of adenosine influx in CNT2 injected oocytes by 100µM concentration of different BAs (pH-7.4). Bars represent Mean ± SD (n=8). ****, p 0.001 (One way ANOVA).

**A**

**B**

**C**

**D**

**Sup Fig2: BAs were incapable of inhibiting nucleoside analogs transport mediated by ∆36ENT3 and ENT2:** The transport activities of different nucleosides by H_2_O, ∆36ENT3 and ENT2 transcripts injected *Xenopus* oocytes in the absence or presence of different BAs. Uptake of ^3^H-AZT and ^3^H-ddI into oocytes at 37^º^C after 24 hrs of injection of transcripts was measured in buffers with different pH for different nucleoside transporters. (A) Inhibition of AZT influx in H_2_O and ∆36ENT3 injected oocytes by 100µM concentration of different BAs (pH-5.5). (B) Inhibition of ddI influx in H_2_O and ∆36ENT3 injected oocytes by 100µM concentration of different BAs (pH-5.5). (C) Inhibition of AZT influx in H_2_O and ENT2 injected oocytes by 100µM concentration of different BAs (pH-5.5). (D) Inhibition of ddI influx in H_2_O and ENT2 injected oocytes by 100µM concentration of different BAs (pH-5.5). Bars represent Mean ± SD (n=8). ****, p 0.001 (One way ANOVA).

**A**

**B**

**D**

**C**

**Sup Fig 3: BAs do not interfere with transport of nucleosides except Ado:** (A-D) Uptake of ^3^H-nucleosides in the presence or absence different BAs (100 µM) in sodium free buffer A) ^3^H-guanosine (0.02 µM). B) ^3^H-cytidine (0.02 µM). C) ^3^H-uridine (0.02 µM). D) ^3^H-thymidine (0.02 µM). Bars represent Mean ± SD (n=3). ****, p 0.001 (One way ANOVA).
